# The Role of Chronic Endometritis in Endometriosis: A Personalized Diagnostic Tool?

**DOI:** 10.3390/jpm16020073

**Published:** 2026-01-31

**Authors:** María Luna Arana, Augusto Pereira Sánchez, Gema Vaquero Argüello, Eva Tejerina González, Milagros Alonso-Iniesta, Tirso Pérez Medina

**Affiliations:** Puerta de Hierro University Hospital, Autonomous University of Madrid, 28049 Madrid, Spain; augusto.pereira@uam.es (A.P.S.); etejegon@telefonica.net (E.T.G.); tirso.perez@uam.es (T.P.M.)

**Keywords:** chronic endometritis, adenomyosis, endometriosis

## Abstract

(1) **Background**: Endometriosis and chronic endometritis (CE) are pathologies that are positively correlated and have similar paracrine and immunological alterations. This leads us to wonder whether their interrelationship plays a role in the etiopathogenesis or progression of endometriosis. The purpose of this study is to evaluate whether patients with endometriosis and CE have a more advanced stage of the disease, higher rates of pain, a poorer response to treatment, and a greater association with other pathologies compared to women with endometriosis without CE. (2) **Methods**: This is a cross-sectional pilot design study of 37 women with endometriosis who underwent endometrial aspiration for the diagnosis of CE and were followed up at the Puerta de Hierro Hospital. (3) **Results**: All patients with CE in this study had adenomyosis (*p* = 0.004). There was a relatively homogeneous distribution of CE in the different endometriosis phenotypes. The group of patients with endometriosis and CE indicated higher rates of pain during ovulation and less pain during defecation and sexual intercourse. (4) **Conclusions**: A high prevalence of CE was observed in patients with endometriosis, as well as a trend suggesting a relationship between CE and adenomyosis that should be studied. The following article attempts to reflect a link found between endometriosis and chronic endometritis, which would be important when prescribing personalized medicine, as it forces us to look for a specific disease in a specific patient profile.

## 1. Introduction

Both endometriosis and chronic endometritis (CE) are fairly prevalent conditions in women, whose symptoms, when present, include infertility and pelvic pain.

The estimated rate of CE in the general population is difficult to define due to the lack of characteristic clinical manifestations and standardized diagnostic criteria, with prevalence rates ranging from around 2.8% [1] and 20% [2] to 60–66% [3] in women with repeated miscarriages or implantation failures, with the actual prevalence remaining unknown to this day. Several factors contribute to the variability in prevalence estimates. These include the heterogeneity of endometrial sampling techniques, differences in histopathological interpretation, and reliance on various diagnostic modalities such as hysteroscopy, culture, and immunohistochemistry. The true prevalence may be underestimated in routine clinical practice due to the focal and sometimes subtle nature of histological changes. Kitaya et al. [4] conducted a study aimed at clarifying the prevalence of chronic endometritis, analyzing the presence of chronic endometritis in 234 patients who underwent hysterectomy. Chronic endometritis was identified in 11% of endometrial samples.

The actual prevalence of endometriosis remains a mystery but is estimated to be around 10% of women of reproductive age [5].

Both conditions are chronic inflammatory diseases with unclear pathogenesis that can cause abnormal uterine bleeding, pain, and reproductive disorders. In addition, they share similar paracrine and immunological alterations, with a proven positive relationship between the two [6,7,8,9], suggesting the existence of a common predisposing factor for both conditions.

One of the most widely accepted theories on the etiopathogenesis of endometriosis is “retrograde menstruation” [10]. However, there is a general consensus that this factor alone is not sufficient and that other phenomena are necessary to cause these regurgitated endometrial fragments to escape immune elimination and become invasive lesions, thus generating chronic inflammation. One of the factors that could be involved in this invasion of regurgitated endometrial tissue could be the theory of bacterial contamination [11], which states that certain bacteria, such as *E. coli*, when present at higher levels in menstrual blood, release endotoxins that activate specific receptors in immune cells, such as macrophages (the main immune cell in endometriosis), capable of triggering a prolonged and sustained inflammatory cascade response. Similarly, higher levels of endotoxins have been observed in menstrual and peritoneal fluids in women with endometriosis [12].

The uterine cavity is connected to the pelvic cavity through the fallopian tubes [7]. Therefore, there may be a bidirectional passage of cells and humoral factors between the two cavities. This would suggest that some humoral factors or substances produced by endometriosis in the pelvic cavity can penetrate the endometrial cavity and induce plasma cell infiltration, leading to CE. In contrast, abnormal plasma cells within the eutopic endometrium can flow into the pelvic cavity with the endometrium shed during menstruation, which could cause or maintain endometriosis. This communication between the endometrial cavity and the pelvic cavity could be the relationship between the two and the reason why both pathologies share so many common pathophysiological characteristics. If we understand this communication, as well as the entire inflammatory, hormonal, and infectious network that exists in both diseases, we could even think that they could be the origin or consequence of each other.

All of this leads to the hypothesis that, in patients with infectious CE, retrograde menstruation or the presence of contaminated blood could serve as a causative or trigger factor for endometriosis. In other words, it could be that the microbiota in a dysbiosis state contributes to immune activation that reinforces and prolongs peritoneal inflammation and, possibly, the progression of endometriosis. This could explain, at least in part, why retrograde menstruation affects some women and not others.

Based on the hypothesis that retrograde menstruation in patients with CE could lead to an increase in endotoxin secretion, thus generating more prolonged and sustained inflammation, our objective is to evaluate in the future whether patients with endometriosis and CE have a higher degree of disease involvement, a more advanced stage, greater associated pain, a poorer response to hormonal treatment, and a greater association with other pathologies compared to women with endometriosis without CE. This allows us to conduct a study and provide personalized treatment according to the patient’s clinical characteristics.

## 2. Materials and Methods

### 2.1. Study Population and Participant Enrollment

This is a cross-sectional pilot design study of 37 women being monitored for endometriosis at the Puerta de Hierro University Hospital from January 2025 to October 2025.

This study is a pilot study for a doctoral thesis, the purpose of which is to test the feasibility of these methods and procedures before conducting a larger study. It does not seek to answer research questions; rather, it seeks to avoid costly mistakes. It serves to evaluate recruitment, retention, measurement instruments, data collection, confounding variables, and staff training. It allows us to test the acceptability, safety, and fidelity of the interventions but not to demonstrate the efficacy.

We define endometriosis as a complex benign gynecological disease characterized by the ectopic presence of endometrial epithelium and stroma outside the uterine cavity [13]. Fifty-four percent of our patients had a histological diagnosis of endometriosis. The remaining 46% were diagnosed with endometriosis by ultrasound and magnetic resonance imaging according to the IDEA consensus [14]. CE is defined as localized inflammation of the endometrial mucosa, characterized by the presence of edema, increased stromal cell density, dissociated maturation between epithelial cells and fibroblasts, and the presence of plasma cells in the stroma [15]. Like other authors we used a count of ≥5 (CD138-positive cells) plasma cells per high-power field as the histological criterion for the diagnosis of CE [16,17]. We use the MUSA [18,19] diagnostic criteria for the ultrasound diagnosis of adenomyosis, always requiring the presence of at least one direct criterion for diagnosis.

The first phase of this study was conducted in accordance with the Declaration of Helsinki and was approved by the Ethics Committee of Hospital Puerta de Hierro (protocol code: PI 42/23, date of approval). The second phase of this study was also conducted in accordance with the Declaration of Helsinki and was approved by the Ethics Committee of Hospital Puerta de Hierro (protocol code: PI 70/25, 30 July 2025, of approval) for studies involving human subjects. Women who wished to participate and met the inclusion criteria underwent endometrial aspiration with a Cornier cannula for the diagnosis of chronic endometritis in the follicular phase in patients with a natural cycle or at any time in patients undergoing hormone treatment. Based on this criterion, two comparative groups were established: women with endometriosis with or without CE.

### 2.2. Inclusion and Exclusion Criteria

Women of reproductive age with a histological diagnosis or imaging tests (ultrasound or magnetic resonance according to the IDEA consensus diagnoses) for endometriosis were included in this study. Patients who had undergone hysterectomy, hysteroscopy, or had taken antibiotics in the 3 months prior to the histological sample were excluded from the study, as were those with submucosal fibroids or endometrial polyps, those undergoing treatment with anticoagulants, or those with coagulation disorders. Women who were menopausal or whose sample was insufficient for the diagnosis of chronic endometritis were excluded.

### 2.3. Variables

The following demographic variables were collected: family history of endometriosis, age, parity, age at menarche, country of origin. The following clinical variables were collected: endometriosis phenotype, presence or absence of adenomyosis, type of adenomyosis (focal or diffuse), previous endometriosis surgery, presence or absence of associated autoimmune disorders, presence or absence of intermenstrual bleeding, number of days of bleeding per month, and visual analog scale (VAS) scores for dysmenorrhea, dyspareunia, pain during urination, and pain during defecation. The following therapeutic variables were also collected: current hormonal treatment for endometriosis and type of hormonal treatment received.

The data were obtained through patient surveys, as well as medical records and hospital databases.

Table 1 presents a numerical description of these variables.

### 2.4. Statistical Analysis

Descriptive statistics are expressed as frequencies and percentages for categorical variables and as mean values with standard deviations for continuous variables. Differences between categorical variables were assessed using Chi-square tests, while the Wilcoxon signed-rank test for independent samples was applied to continuous data given the limited sample size.

All values of *p* < 0.05 were considered statistically significant.

To investigate the association between CE and selected explanatory variables, a multivariable logistic regression model was constructed after evaluating collinearity among covariates. A Bayesian framework was adopted instead of the conventional frequentist approach for several reasons. The relatively small sample size may reduce the reliability of maximum likelihood estimates and widen confidence intervals, while Bayesian methods allow the incorporation of weakly informative priors. In addition, Bayesian inference provides full posterior distributions of the parameters, enabling direct probability statements regarding associations, which are often more clinically relevant than binary hypothesis testing.

Model selection was based on the Deviance Information Criterion (DIC), with lower values indicating better fit while penalizing overparameterization. Vague prior distributions were specified for the fixed effects (Gaussian prior with mean 0 and precision 0.001). Model estimation was performed using the Integrated Nested Laplace Approximation (INLA) method, which was chosen for its computational efficiency and accuracy compared with traditional Markov Chain Monte Carlo (MCMC) techniques in approximating posterior distributions.

Statistical analyses were performed using R Statistical Software (version 4.4.2) and the stable version of R-INLA INLA_24.12.11 [20,21].

## 3. Results

The initial sample included 37 patients, of whom 7 were excluded because their endometrial samples were not representative, leaving 30 patients for analysis (18 with chronic endometritis and 12 without).

### 3.1. Descriptive Analysis

The mean age was 36.8 years (age range between 21 and 48), with no significant differences between women with or CE. In our population, the age range of women who tested positive for chronic endometritis ranged from 21 to 48 years old, with a mean age of 36 years. The age range of patients who tested negative for chronic endometritis ranged from 25 to 44 years old, with a mean age of 36 years in this population as well. Similarly, parity and endometrioma size did not differ significantly between groups. The average number of bleeding days was slightly lower in women with CE (5.3 ± 3.9) compared to those without (6.2 ± 3.7), though this difference was not statistically significant.

Table 1 summarizes the clinical and demographic characteristics of the study population.

Regarding pain, VAS scores were generally comparable. Dysmenorrhea was intense across both groups (mean score: 8.1; range: 4–10), while dyspareunia appeared higher in women without CE (4.2 ± 2.4) than in those with CE (2.6 ± 3.5), without reaching statistical significance (*p* = 0.163). Pain during urination was minimal in both groups, while pain during defecation was lower in patients with CE (2.4 ± 3.5 vs. 4.4 ± 3.7, *p* = 0.107). Pain during ovulation showed no significant differences.

Most patients reported no family history of endometriosis (83.33%), and the distribution was identical in both groups. Previous surgeries were observed in 11 patients, without significant differences (*p* = 1). Autoimmune disorders were rare (five cases overall—16.67%) and showed no differences.

**Figure 1:**
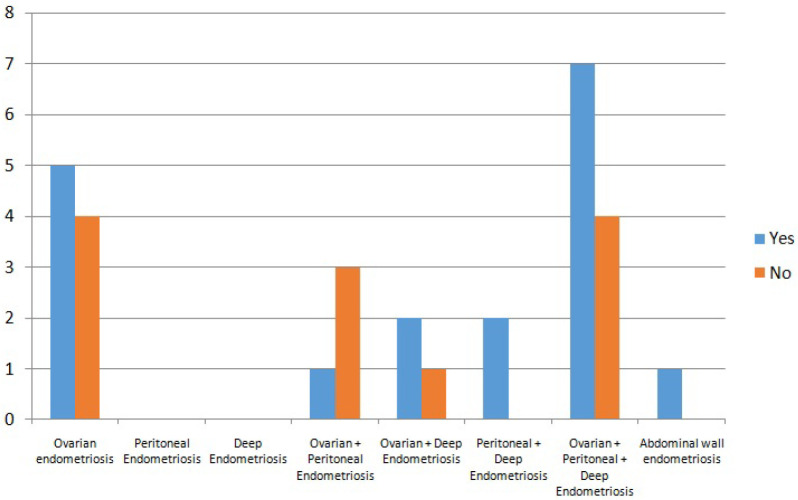
Endometriosis phenotypes included.

Endometriosis is categorized into 4 subtypes [22]: superficial peritoneal, deep, ovarian (endometriomas), and extrapelvic endometriosis. Subtypes may occur alone or in combination. In our study endometriosis phenotypes were heterogeneously distributed (as can be seen in the Figure 1), with ovarian and mixed phenotypes being the most common (90%), but without association with CE (*p* = 0.69). When analyzed individually, ovarian involvement is the only endometriosis phenotype that comes somewhat closer to statistical significance in terms of the presence of chronic endometritis, but without actually reaching it. Perhaps with a larger sample size it would be significant.

Seventeen women were receiving hormonal treatment, with similar proportions in both groups (*p* = 1). The type of treatment varied, most commonly oral contraceptives or progesterone-only pills, without significant differences. Intermenstrual bleeding was more frequent in women without CE, though not significantly (*p* = 0.210). All patients receiving hormone treatment did so because of painful symptoms. Women who did not receive hormone treatment did so because they wanted to have children or because they did not want hormone treatment.

Adenomyosis showed a strong association with CE: all women with CE had adenomyosis, compared with only five in the group without CE (*p* = 0.004). Among them, diffuse adenomyosis predominated, while focal adenomyosis was only observed in women without CE (*p* = 0.039), establishing a odds ratio risk of developing CE in patients with endometriosis without adenomyosis of 0 (0, 0). Those values of 0 (0, 0) come from a covariate that does not present cases of chronic endometritis in one of the values. Therefore, the model lacks sufficient information to accurately identify the size of the effect, generating very high coefficients, which, when the exponential of the logistic regression is undone, generates values that are, for practical purposes, 0, although certainly not absolute 0.

### 3.2. Modelization

A multivariable logistic regression was performed to evaluate the association between CE and several explanatory variables of clinical interest. Based on the descriptive study, only those covariates that appeared to have some effect and could be potentially relevant were selected. 11 candidate variables were considered for inclusion. Specifically, the following were tested: age, age at menarche, ovarian endometriosis, presence of adenomyosis, parity, presence of associated pain symptoms, hormonal treatment with IUD, degree of pain improvement, and intermenstrual bleeding.

Before model selection, correlations between covariates were assessed to avoid collinearity. The highest correlation was observed between parity and adenomyosis (r = 0.41)—a value not considered sufficiently high to justify exclusion. Consequently, all variables were retained for testing. To identify the best-fitting model, and given the low computational cost of our chosen estimation strategy, all possible combinations of covariates (511 models in total) were compared using the Deviance Information Criterion (DIC), a penalized measure that rewards parsimony. Lower DIC values indicate better statistical performance.

The best-fitting model included the covariates of age, the presence of pain symptoms, the presence of adenomyosis, ovarian endometriosis, and hormonal treatment with IUD, yielding a DIC of 58.58. Odds ratios were not reported due to the small sample size, which produces extreme and unstable values when coefficients are exponentiated. Instead, the results should be interpreted in terms of potential risk factors (posterior probability is above zero) and protective factors (posterior probability is below zero).

The presence of pain symptoms and hormonal treatment with IUD showed strong positive associations with CE, with posterior probabilities close to or exceeding 99%. In contrast, the absence of adenomyosis and ovarian endometriosis acted as protective factors, both with negative coefficients and low posterior probabilities. Increasing age also showed a modest protective effect. Taken together, these findings suggest that adenomyosis, ovarian disease, and specific hormonal treatments may play an important role in the occurrence of CE in women with endometriosis, although the limited sample size warrants cautious interpretation. It is important to remember, given the retrospective nature of the study and the possibility of unbalanced data, the potential instability of the models, and it is inadvisable to interpret these coefficients as strong effects.

Table 2 summarizes the regression coefficients.

## 4. Discussion

In this study, we detected a positive correlation between endometriosis, chronic endometritis, and adenomyosis.

CE is defined as localized inflammation of the endometrial mucosa, characterized by the presence of edema, increased stromal cell density, dissociated maturation between epithelial cells and fibroblasts, and the presence of plasma cells in the stroma [15]. CE is the result of various infectious and non-infectious factors that cause persistent inflammation of the endometrium. The pathogenesis appears to be related to a qualitative and quantitative alteration of the endometrial microbiome, which leads to the abnormal proliferation of different types of microorganisms, mainly Gram-negative and intracellular bacteria [2,23], generating an immune-mediated response, whose main characteristic is the infiltration of plasma cells, which are rarely found in the endometrium outside this scenario. Histological diagnosis in the follicular phase, through immunohistochemical identification with the CD138 of plasma cells in the endometrial stroma, is currently the gold standard for the diagnosis of CE. A recent meta-analysis examining the correlation between plasma cell count and reproductive outcomes found a significant association between spontaneous abortion rates and plasma cell counts greater than 5 per high-power field. Based on these findings, a threshold of ≥5 plasma cells/10 HPF may be more appropriate for diagnosing CE [24]. Inducing a chronic state of inflammation ideal for disease progression [25].

Endometriosis is defined as a complex benign gynecological disease characterized by the ectopic presence of endometrial epithelium and stroma outside the uterine cavity. Bleeding from these ectopic implants leads to local inflammation responsible for the formation of adhesions and fibrosis, which, in some cases, will produce symptoms such as severe pelvic pain, dysmenorrhea, dyspareunia, dyschezia, and subfertility [13]. Endometriosis is a multifactorial disease whose etiology and pathogenesis remain unknown. One of the most widely accepted theories about the origin of ectopic endometrial tissue is that of “retrograde menstruation” [10]. However, most women experience retrograde menstruation [26], so other mechanisms are necessary to facilitate the development of endometriosis. There must be other factors that allow these regurgitated endometrial remnants to escape immune elimination, adhere to the peritoneal epithelium, invade it, establish local neurovascularization, and continue to grow and survive, thus becoming invasive lesions [27,28].

By understanding the pathophysiology of both diseases individually and since the uterine cavity is connected to the pelvic cavity through the fallopian tubes, so cells and humoral factors can pass between the cavities, we can begin to find similarities between them, which lead us to suspect an increasingly apparent correlation between the two.

In both diseases, inflammation and the immune-mediated response represent a key aspect of their pathophysiology. Several studies, which we will detail below, have already shown that endometriosis and CE share common immunological backgrounds. For example, in both diseases, unusual infiltration of B cells and plasma cells in the endometrium has been documented, along with increased local production of several proinflammatory cytokines, such as IL-6 and TNF-α [29,30]. These elevated inflammatory responses may be potentially related to the progression and development of both diseases.

In addition, the gene expression levels of molecules associated with proliferation are abnormally upregulated in the secretory phase endometrium with CE [31], indicating that the endometrium with CE is unable to respond adequately to progesterone. This indicates that CE and endometriosis share common characteristics in terms of endometrial resistance to progesterone [32].

On the other hand, as in CE, in endometriosis the microbiota could also play an important role. The microbiota in a state of dysbiosis would alter normal immune function, causing aberrant inflammatory responses by elevating proinflammatory cytokines, compromising immune surveillance, and altering immune cell profiles [11,12]. Thus, this dysbiosis, with the consequent secondary immune dysregulation that appears, could generate a chronic state of inflammation ideal for the onset and progression of both pathologies, if they are not a sequence of the same.

Pinto et al. [33] also hypothesized that the alteration in uterine contractility caused by CE may induce retrograde menstruation, which could be a possible etiopathogenic link between the two conditions. In women with CE, a different pattern of uterine contractility was observed during the different phases of the menstrual cycle compared to women without CE. In particular, a decrease in antegrade subendometrial contractions present during menstruation was observed, facilitating retrograde reflux of menstrual blood through the fallopian tubes. This leads us to speculate that, according to Sampson’s theory, CE could represent a facilitating factor in the development of endometriosis.

In turn, heavy menstrual bleeding, short menstrual cycles, and prolonged exposure to endogenous estrogen, symptoms [34,35] associated with chronic endometritis, could be other factors involved in the interrelationship between endometriosis and chronic endometritis.

An increasing number of publications have revealed an association between CE and endometriosis [6,7,8,9].

In 2014, Takebayashi et al. [7] found that women with endometriosis had a higher rate of concomitant CE than those without endometriosis (52.9% vs. 27.0%), regardless of disease stage. It was also observed that the association of the number of plasma cells per high-power field was higher in patients with endometriosis. Two suggestions were made: first, that endometriosis was a significant predictor of CE, and second, that the degree of inflammation or severity of CE was higher in patients with endometriosis.

In turn, this group analyzed the rate of CE at each stage of endometriosis. The rates did not correlate with the stage, and deep endometriosis lesions were not related to CE. These results suggested that EC could be an independent complication of endometriosis or be involved in the pathogenesis of endometriosis, since EC appeared even in stage I. This coincides with the data obtained in our study, which found no statistical significance between CE and the stage or degree of endometriosis involvement. However, in our study, it appears that there may be some relationship between ovarian phenotype endometriosis and the presence of CE. The *p*-value was 0.25, which is not sufficient to confirm the existence of evidence, but perhaps with a larger sample size, we would be able to detect statistically significant evidence.

This could coincide in part with what was published by Lin et al. [8], those who corroborate that the incidence rate of CE was slightly lower in patients with peritoneal endometriosis compared to those with ovarian endometriosis, although these differences were not statistically significant.

Subsequently, Cicinelli et al. [36], in a retrospective analysis of a total of 156 patients who underwent hysterectomy, demonstrated that CE coexists in approximately 40% of cases of endometriosis. However, they found no significant association between CE and age, BMI, and the presence/absence of uterine fibroids and/or adenomyosis.

Later, in 2024, Li et al. published a retrospective study [9] that aimed to explore the relationship between intrinsic and extrinsic adenomyosis and CE. In this study, patients with internal adenomyosis had the lowest incidence of CE. In addition, the incidence of CE was found to be influenced by the type of adenomyosis, as indicated by multivariate logistic regression analysis. Compared to patients with internal adenomyosis, those with full-thickness adenomyosis had a significantly higher rate of CE.

The role played by adenomyosis in this study remains unknown. The relationship between endometriosis and adenomyosis has been the subject of study over the last few decades. This narrative review [37] explored all aspects of endometriosis and adenomyosis. Although many aspects of these conditions are well documented, there is still no comprehensive framework that takes into account their frequent coexistence and overlapping symptoms. We observed that adenomyosis is more frequently seen in women with endometriosis and infertility, with prevalence rates ranging from 35% to 79%. As with endometriosis, women with adenomyosis show a greater response to estrogen as well as resistance to progesterone. One of the most common symptoms of adenomyosis is chronic pelvic pain as well as heavy menstrual bleeding or menorrhagia. It can also increase the risk of infertility by interfering with embryo implantation. These similarities between the two conditions are quite intriguing. The possible relationship between chronic endometritis and adenomyosis suggested by our study remains to be elucidated.

Our study found a correlation between adenomyosis and CE. Multivariate statistical analysis found a high probability of correlation between the two, but this must be confirmed with a larger sample size. This pilot study allows us to justify the need to increase the sample size and adjust for confounding factors in order to draw definitive conclusions. We have detected a trend that suggests big correlation between CE and the variable presence of adenomyosis, that must be proven, with this correlation being greater in patients with diffuse adenomyosis. This could be of interest in the future when looking for this pathology in this specific patient profile. It remains to be investigated whether selective treatment of these patients could benefit the course of endometriosis or improve the symptoms derived from both pathologies. A hypothetical explanation for this could be that dysbiosis occurring in EC could influence damage to the endometrial interface, favoring the development of adenomyosis. Further studies are needed to answer these questions.

On the other hand, in our study, there appears to be other association between CE and parity, CE and IUD users, CE and the presence of intermenstrual bleeding, and CE and higher pain scores with ovulation. All of these have p-values greater than 0.05 but close to it; with a larger sample size, the association will probably be found to be statistically significant. This would also lead us to investigate this disease in this selective group of patients. One possible explanation for the association between CE and IUDs could be that the presence of a foreign body in the endometrial cavity could promote the microenvironment necessary for the development of CE. One possible explanation for the increased frequency of intermenstrual bleeding associated with EC could be due to the added inflammation of the endometrium that occurs in these women, or even, who knows, perhaps combined adenomyosis could explain or play a role in this symptom. Further studies are needed to answer these questions.

Among the limitations of this study, in addition to it being a non-randomized study, the small sample size stands out, which may lead to erroneous associations or a lack thereof. In turn, the lack of knowledge about the prevalence and lack of established diagnostic criteria for chronic endometritis may lead to overdiagnosis. The diagnostic method for CE used in this study may be a false positive due to contamination or other unknown factors. Chronic endometritis is a reversible cause of infertility and remains a clinical and biological challenge even when using conventional hysteroscopy and histology. This study is based on data published by Ilic et al. [38], which highlight the possible low accuracy or relevance of hysteroscopy. And from studies conducted by Morento et al. [39], which concluded that the combined use of histology, hysteroscopy, and microbial culture for the diagnosis of chronic endometritis significantly increased complexity and cost, and consistent and concordant results were obtained in only 20% of cases of chronic endometritis. However, the concomitant use of hysteroscopy could be helpful and play a role, especially in women with concomitant adenomyosis.

Another diagnostic limitation is the timing of sampling. The phase of the menstrual cycle in which the biopsy is performed may be important, as may whether hormone treatment influences the diagnosis. According to the literature [40], during the secretory phase, plasma cells are only present in the basal stromal layer, which would cause them to go unnoticed if they were not included in the biopsy. In our study, patients who were not undergoing hormone treatment (43.33%) underwent aspiration during the proliferative phase. However, 56.67% of patients were undergoing hormone treatment. We do not have studies reporting the ideal time for endometrial sampling in patients using hormonal contraception. On the other hand, since endometriosis is such a complex disease, with unknown etiopathogenesis, certain variables in this study could act as confounding factors (e.g., IUD use as a source of endometrial inflammation). Furthermore, adenomyosis is almost universal in the CE group, suggesting a possible selection bias. In addition, patients were recruited from a tertiary referral center, which may increase the presence of serious diseases and adenomyosis, and generate selection or diagnostic bias. To minimize this effect, stratified recruitment or matching according to adenomyosis status would help to unravel these effects. These limitations can probably be corrected in the future by increasing the sample size and performing a subgroup analysis.

One variable that could be important to collect is the history of infertility or sterility in patients to avoid possible confounding factors in the analysis of results. However, we found the following meta-analysis [41], which showed no significant differences between the two groups; the combined prevalence of CE in women with infertility and endometriosis was 19%.

Despite the limitations described, these suggested data could lead us to more personalized and individualized medicine, in which targeted diagnostic and therapeutic strategies are offered based on the characteristics of the selected population. Knowing this data and developing it could lead to the implementation of a selective screening strategy for chronic endometritis in patients with endometriosis who also have adenomyosis, or who use hormonal IUDs or have associated symptoms such as intermenstrual bleeding. This would enable more personalized medicine for patients with endometriosis.

These data invite us to conduct a more comprehensive study to draw definitive conclusions and determine more precisely the relationship between endometriosis, chronic endometritis, and adenomyosis. We also aim to identify possible risk factors for CE, such as adenomyosis or use of an intrauterine device. These data also invite us, for example, to investigate the role of hormone treatment in the diagnosis of chronic endometritis, whether endometriosis pain rates change after antibiotic treatment of CD, or the role of hysteroscopy in this scenario.

## 5. Conclusions

Our study observes a high prevalence of CE in patients with endometriosis and a trend that suggests a big relationship between CE and adenomyosis, with a higher risk between both pathologies. This study did not find a more advanced stage of the disease or a worse response to treatment in women with endometriosis and CE compared to women without CE, but a larger sample size is needed. Patients with endometriosis and CE, it is suggested, were associated with a higher risk of intermenstrual bleeding, higher levels of ovulatory pain, and lower levels of pain during defecation and sexual intercourse, without reaching statistical significance, probably due to the sample size. There also appeared to be a higher probability of CE prevalence in patients using intrauterine devices. The discovery of chronic endometritis in patients with endometriosis and other associated clinical factors would allow us to specifically target chronic endometritis, treat it specifically, and see if we can modulate the course of endometriosis. Could these findings lead to more personalized medicine for patients with endometriosis, with more targeted treatment?

This study is a pilot trial designed to study the preliminary relationship between both pathologies, with the aim of determining whether a more extensive study is needed to draw definitive conclusions. Furthermore, given the similar pathophysiology and findings, it seems logical to rule out the presence of CE in symptomatic women with endometriosis and we believe it may be useful to publish these preliminary results in order to broaden the scope of research and raise new questions, relationships, diagnostic and therapeutic strategies.

## Figures and Tables

**Table 1:** Numerical description of the variables included. Data are presented as mean ± standard deviation or n (%). Significance set at *p* < 0.05 *, *p* < 0.01 **, respectively.

Characteristics	All (n = 30)	CE (18)	No CE (12)	*p*-Value
Age	36.77 ± 6.28	36.89 ± 6.95	36.58 ± 5.40	0.894
Age of menarche	12.87 ± 1.72	12.78 ± 1.86	13.00 ± 1.54	0.683
Parity	0.6 ± 0.81	0.78 ± 0.88	0.88 ± 0.33	0.122
Endometrioma size	3.30 ± 1.94	3.00 ± 1.56	3.67 ± 2.35	0.673
Days of bleeding	5.70 ± 3.80	5.33 ± 3.96	6.25 ± 3.65	0.315
VAS for dismenorrhea	8.13 ± 1.81	8.06 ± 1.89	8.25 ± 1.76	0.794
VAS for dispareunia	3.20 ± 3.16	2.56 ± 3.50	4.17 ± 2.37	0.163
VAS for pain during urination	1.60 ± 2.74	1.33 ± 2.61	2.00 ± 2.98	0.583
VAS for pain when defecating	3.23 ± 3.65	2.44 ± 3.48	4.42 ± 3.70	0.107
VAS for pain on ovulation	5.83 ± 3.15	6.22 ± 3.00	5.25 ± 3.41	0.894
Pain improvement after treatment	0.60 ± 0.29	0.65 ± 0.31	0.53 ± 0.26	0.172
**Family history of endometriosis**				
Yes	5 (16.67%)	3 (16.67%)	2 (16.67%)	1
No	20 (83.33%)	15 (83.33%)	10 (83.33%)	
**Previous Surgeries**				
Yes	11 (36.67%)	7 (38.89%)	4 (33.33%)	
No	18 (60%)	11 (61.11%)	7 (58.33%)	1
NA (Not correctly determined)	1 (3.33%)	0 (0%)	1 (8.33%)	
**Type of Endometriosis**				
Ovarian	9 (30%)	5 (27.78%)	4 (33.33%)	
Ovarian + Peritoneal	4 (13.33%)	1 (5.56%)	3 (25%)	
Ovarian + Deep	3 (10%)	2 (11.11%)	1 (8.33%)	0.696
Peritoneal + Deep	2 (6.67%)	2 (11.11%)	0 (0%)	
Ovarian + Peritoneal + Deep	11 (36.67%)	7 (38.89%)	4 (33.33%)	
Abdominal Wall	1 (3.33%)	1 (5.56%)	0 (0%)	
**Associated Autoimmune Disorders**				
Yes	5 (16.67%)	2 (11.11%)	3 (25%)	0.364
No	25 (83.33%)	16 (88.89%)	9 (75%)	
**Hormonal Treatment**				
Yes	17 (56.67%)	10 (55.56%)	7 (58.33%)	1
No	13 (43.33%)	8 (44.44%)	5 (41.67%)	
**Intermenstrual Bleeding**				
Yes	8 (26.67%)	3 (16.67%)	5 (41.67%)	0.210
No	22 (73.33%)	15 (83.33%)	7 (58.33%)	
**Type of hormonal treatment**				
COC	8 (26.67%)	5 (27.78%)	3 (25%)	
COC + POCs	10 (33.33%)	4 (22.22%)	6 (50%)	
POCs	3 (10%)	1 (5.56%)	2 (16.67%)	
POCs + Hormonal IUD	2 (6.67%)	2 (11.12%)	0 (0%)	0.593
COC + Hormonal IUD	1 (3.33%)	1 (5.56%)	0 (0%)	
(Other)	2 (6.67%)	2 (11.12%)	0 (0%)	
NA (Not correctly determined)	4 (13.33%)	3 (16.67%)	1 (8.33%)	
**Associated Pain**				
Yes	8 (26.67%)	3 (16.67%)	5 (41.67%)	0.210
No	22 (73.33%)	15 (83.33%)	7 (58.33%)	
**Presence of Adenomyosis**				
Yes	23 (76.67%)	18 (100%)	5 (41.67%)	0.004 **
No	7 (23.33%)	0 (0%)	7 (58.33%)	
**Type of Adenomyosis**				
Focal	2 (6.67%)	0 (0%)	2 (16.67%)	0.039 *
Diffuse	21 (70%)	18 (100%)	3 (25%)	
No Presence	7 (23.33%)	-	-	

Abbreviations: CE: chronic endometritis; VAS: visual analog scale; Deep: deep endometriosis; COC: combined oral contraceptives; POCs: progestogen-only contraceptives; IUD: intrauterine device.

**Table 2:** Numerical description of the results obtained from the multivariable logistic regression.

Variable	Regression Coefficient	Probability > 0
Age	−0.3 (−0.56, −0.04)	1.26
Pain Associated (No)	16.29 (13.46, 19.12)	100
Presence of Adenomyosis (No)	−28.83 (−49.15, −8.52)	0.27
Endometriosis–Ovaric (Yes)	−37.44 (−59.86, −15.01)	0.05
Hormonal Treatment IUD (Yes)	28.88 (4.63, 53.14)	99.02

Abbreviations: IUD: intrauterine device.

## Data Availability

The dataset presented in this article is not readily available to preserve patient privacy. Access to the dataset may be granted upon reasonable request to the corresponding author, subject to institutional review and data sharing agreements.

## Abbreviations

The following abbreviations are used in this manuscript:

CE	Chronic endometritis
VAS	Visual analog scale
IUD	Intrauterine device
